# Learning as you go: an audit of continuing professional development opportunities in the workplace

**DOI:** 10.1186/1757-1146-4-S1-P49

**Published:** 2011-05-20

**Authors:** Katrina Richards

**Affiliations:** 1 Podiatry Department, Eastern Health, Australia

## 

The introduction of mandatory Continuing Professional Development (CPD) as a condition of podiatry registration has caused quite a deal of manic amongst the Podiatry population. What practitioners may underestimate is the amount of CPD that is undertaken as part of their everyday work practices. The podiatrists at Eastern Health are fortunate to have a supportive work environment that aims to foster learning opportunities and career advancement. This poster shows an audit of the amount and types of learning opportunities logged by the 12 member podiatry department for the first six months of the mandatory CPD period. The audit uncovered that many of the staff members had satisfied the Podiatry Board of Australia’s mandatory requirements within a two-month period. It is hoped that this poster will inspire others to think about their ongoing CPD opportunities within their own workplace.

